# Clinical and genetic factors associated with increased risk of severe liver toxicity in a monocentric cohort of HIV positive patients receiving nevirapine-based antiretroviral therapy

**DOI:** 10.1186/s12879-018-3462-5

**Published:** 2018-11-12

**Authors:** Andrea Giacomelli, Agostino Riva, Felicia Stefania Falvella, Maria Letizia Oreni, Dario Cattaneo, Stefania Cheli, Giulia Renisi, Valentina Di Cristo, Angelica Lupo, Emilio Clementi, Stefano Rusconi, Massimo Galli, Anna Lisa Ridolfo

**Affiliations:** 1 Infectious Diseases Unit, DIBIC Luigi Sacco - University of Milan, Via G.B. Grassi, 74, 20157 Milan, Italy; 2 ASST Fatebenefratelli-Sacco, Clinical Pharmacology Unit, Milan, Italy; 3 E. Medea Scientific Institute, Bosisio Parini, Italy

**Keywords:** Nevirapine, Pharmacogenetic, Hepatotoxicity, ABCB1

## Abstract

**Background:**

Nevirapine has been used as antiretroviral agent since early ‘90. Although nevirapine is not currently recommended in initial anti-HIV regimens, its use remains consistent in a certain number of HIV-1-positive subjects. Thus, our aim was to determine clinical and genetic factors involved in the development of severe nevirapine induced liver toxicity.

**Methods:**

We retrospectively analyzed all HIV positive patients who were followed at the Infectious Diseases Unit, DIBIC Luigi Sacco, University of Milan from May 2011 to December 2015. All patients treated with nevirapine who underwent a genotyping for the functional variants mapping into ABCB1, CYP2B6, CYP3A4 and CYP3A5 genes were included in the analysis. Severe hepatotoxicity was defined as ACTG grade 3–4 AST/ALT increase during the first three months of nevirapine treatment. The causality assessment between NVP exposure and drug-induced liver injury was performed by using the updated Roussel Uclaf Causality Assessment Methods. Hardy Weinberg equilibrium was tested by χ^2^ test. A multivariable logistic regression model was constructed using a *backward* elimination method.

**Results:**

Three hundred and sixty-two patients were included in the analysis, of which 8 (2.2%) experienced a severe liver toxicity. We observed no differences between patients with and without liver toxicity as regards gender, ethnicity, age and immune-virological status. A higher prevalence of HCV coinfection (75.0% vs 30.2%; *p = .0013*) and higher baseline AST (58 IU/L vs 26 IU/L; *p = 0.041*) and ALT (82 IU/L vs 27 IU/L; *p = 0.047*) median levels were observed in patients with liver toxicity vs those without toxicity. The genotypes CT/TT at ABCB1 rs1045642 single nucleotide polymorphism (SNP), showed a protective effect for liver toxicity when compared with genotype CC (OR = 0.18, 95%CI 0.04–0.76; *p = 0.020*) in univariate analysis. In the multivariate model, HCV coinfection was independently associated with higher risk of developing liver toxicity (aOR = 8.00, 95%CI 1.27–50.29; *p = 0.027*), whereas ABCB1 rs1045642 CT/TT genotypes (aOR = 0.10, 95%CI 0.02–0.47; *p = 0.004*) was associated with a lower risk.

**Conclusions:**

According to our findings HCV coinfection and ABCB1 rs1045642 SNP represent independent determinants of severe liver toxicity related to nevirapine. This genetic evaluation could be included as toxicity assessment in HIV-1-positive subjects treated with nevirapine.

## Background

Nevirapine (NVP) is a potent non-nucleoside reverse transcriptase inhibitor widely prescribed in low-income countries for HIV treatment and prevention of mother-to-child transmission of HIV [1]. In high-resource countries, NVP is no longer included among antiretrovirals recommended for initial antiretroviral treatment (ART), although it still remains a valid component of regimens used as ART simplification strategy due to its high efficacy, good metabolic profile, convenience, and low cost [2–4].

Although generally well tolerated and effective, some individuals exposed to NVP develop in the short-term hypersensitivity reactions which can manifest as hepatotoxicity and/or severe cutaneous adverse reactions [5]. Hepatotoxicity, in particular, has been reported more commonly with NVP than with other antiretroviral drugs [6, 7]. Higher baseline and nadir CD4 cell count have been found to independently influence the risk of NVP-related adverse reactions and the use of NVP in *naive* women (with CD4 > 250 cells/uL) and males (with CD4 > 400 cells/uL) is disallowed [8]. The role of the immune system, i.e. higher CD4 cells count, in the development of NVP induced skin and liver toxicity is corroborated by the higher incidence of these events in HIV negative patients receiving NVP as a component of post exposure prophylaxis [5]. Nevertheless, when NVP is used in ART-*experienced* patients with a controlled viremia the risk of development hepatic or cutaneous adverse events significantly decrease and there is no evident association with CD4 cell count [9–11].

A series of demographic and clinical factors have been found to correlate with an increased risk of NVP-toxicity. In particular, risk factors for ALT and AST elevations during NVP therapy included alteration of liver enzymes before NVP start, co-infection with hepatitis B or hepatitis C viruses, female gender and low body weight [6, 12].

A number of studies have also investigated the possible predictive role of genetic polymorphisms of CYP enzymes or drug-transporters involved in NVP metabolism in predisposing to NVP-related adverse effects. NVP is metabolized by cytochrome P450 enzymes CYP2B6 and CYP3A4 with a minor contribution from CYP3A5 [13]. Single nucleotide polymorphisms (SNPs) have been found to impact NVP pharmacokinetics in ethnic mix populations [14–16]. The genotype TT (c.516/rs3745274) in the CYP2B6 gene, in particular, has been associated with higher plasma concentrations of NVP and its possible role in increasing the risk of hepatotoxicity has been hypothesized [14]. However, there is contrasting evidence of interactions between the presence of variant alleles of CYP2B6 and the development of NVP-induced hepatotoxicity [17–19]. The role of CYP3A4 and CYP3A5 variants in determining NVP plasma concentration and the development of liver toxicity is more controversial, with only one report of association between CYP3A5 variants and transaminase values in African patients exposed to NVP [20]. Moreover, although effects of the efflux transporter P-glycoprotein encoded by the ATP Binding Cassette Subfamily B Member 1 (ABCB1) gene on NVP pharmacokinetics remains controversial, two studies have found a protective effect of ABCB1 c.3435 T allele against NVP-related hepatotoxicity [19, 20].

The majority of the studies that evaluated the correlation between pharmacogenetic profiles and NVP-related hepatotoxicity have been performed mainly on African population. However, genetic variant frequencies can differ markedly between different populations and only few data are available on the mentioned pharmacogenetic profiles in non-African populations.

With this in mind, we assessed clinical and pharmacogenetic factors associated with the risk of severe NVP induced liver toxicity in a population of HIV-positive patients attending a clinical center in Italy.

## Methods

This study was conducted on a cohort of adult HIV-positive patients attending the Infectious Diseases Unit, DIBIC Luigi Sacco, University of Milan between May 1 2011 and December 31 2015. Patients who have ever received or were receiving a NVP-containing cART at our clinical center were eligible for the analysis.

Patient’s demographic (age, gender, and ethnicity), epidemiological (HIV acquisition risk) and clinical (CDC stage, body mass index, coinfections, previous and current antiretroviral regimens, immune-virological and hemato-biochemical parameters) data registered during medical visits (on average every three months) are routinely collected in a structured database, allowing the use of the database for clinical, epidemiological or therapeutic studies.

Severe hepatotoxicity was defined as ACTG grade 3–4 AST and/or ALT increase (AST or ALT elevation above 5 time the upper reference limit) during the first three months of nevirapine treatment. The causality assessment between NVP exposure and drug-induced liver injury was performed by using the updated Roussel Uclaf Causality Assessment Methods (RUCAM) [21]. According to the RUCAM, patients were firstly assessed for hepatocellular, cholestatic or mixed liver injury. Subsequently, the score was applied and single cases of NVP-induced liver injury were classified accordingly to the RUCAM total score interpretation and causality grading: ≤0, excluded; 1–2, unlikely; 3–5, possible; 6–8, probable and ≥ 9 highly probable [21].

All patients who met the inclusion criteria underwent a genotyping for the functional variants mapping in ABCB1 (c.3435/rs1045642), CYP2B6 (c.516/rs3745274), CYP3A5 (*3/rs776746) and CYP3A4 (*22/rs35599367) genes. Genomic DNA was isolated from peripheral blood cells using an automatic DNA extraction system (Maxwell® 16 System, Promega) according to the manufacturer’s instructions. All genotypes were determined by Real-Time PCR, using a panel of LightSNiP from TIB-MolBiol (assays based on SimpleProbe®). At the end of the amplification a melting curve analysis was performed (LightCycler 480, Roche).

### Statistical analysis

Baseline clinical characteristics and genotypes of the two groups of interest, i.e. patients who developed severe hepatotoxicity and those who did not, were compared using the χ2 or Fisher’s exact test for categorical variables and the Mann-Whitney test for continuous variables.

Hardy Weinberg equilibrium was tested by χ^2^ test.

The association of clinical and genotypic variables with the development of sever liver toxicity was tested by means of a univariate logistic regression model, and all variables were incorporated into a multivariate logistic regression model with a *backward* elimination method. Statistical significance was defined at 2-sided *P* value < 0.05. The risks were expressed as adjusted odds ratios (aOR) with relative confidence intervals (95% CI). To perform statistical analysis we used the SAS software version 9.3.

The study was reviewed and approved by our ethics committee (Comitato Etico Interaziendale, Milano area 1); all subjects signed a dedicated informed consent.

## Results

A total of 362 patients were included in the analysis. Most of them were ART-*experienced* at the time of initiation of NVP-containing regimen, whereas a minority (16.1%) were ART-*naïve.* Overall 8 (2.2%) patients experienced a severe liver toxicity during the first three months from NVP initiation. Table 1 shows the comparison of patients who experienced a severe hepatotoxicity and those who did not. There was no significant difference as regards gender, ethnicity, age and baseline immune-virological status between the two groups although females showed a trend towards a higher frequency of NVP-induced hepatotoxicity (75.0% vs 35.9%; *p* = 0.055). Conversely, patients who developed severe hepatotoxicity were more frequently HCV-coinfected (75.0% vs 30.2%: *p = 0.013*), showed higher baseline AST and ALT median levels (58 IU/L vs 26 IU/L *p = 0.041,* 82 IU/L vs 27 IU/L *p = 0.047,* respectively), and a lower median baseline body mass index value (19.9 kg/m^2^ vs 22.6 kg/m^2^; *p* = 0.017).

**Table 1:** Baseline characteristics

	Total *n* = 362	Hepatotoxicity *n* = 8	No hepatotoxicity *n* = 354	p*
Age, median (IQR)	38.5 (33.7–45.8)	39.6 (32.7–40.7)	38.5 (33.8–45.9)	0.479
Female, n (%)	133 (36.7)	6 (75.0)	127 (35.9)	0.055
Naïve patients, n (%)	58 (16.0)	1 (12.5)	57 (16.1)	0.783
Risk group, n (%)				
Eterosexual	181 (50.0)	4 (50.0)	177 (50.0)	0.213
MSM	83 (22.9)	0 (0.0)	83 (23.5)	
IVDUs	84 (23.2)	4 (50.0)	80 (22.6)	
Others	14 (3.9)	0 (0.0)	14 (3.9)	
Caucasian, n (%)	330 (91.1)	7 (87.5)	323 (91.2)	0.527
BMI, median (IQR)	22.4 (20.5–24.5)	19.9 (18.3–22.0)	22.6 (20.6–24.5)	*0.017*
AIDS, n (%)	63 (17.4)	1 (12.5)	62 (17.5)	0.999
CD4+/mL, median (IQR)	436 (306–593)	555 (479–611)	433 (300–592)	0.157
HIV-RNA log_10_ cp/mL,median (IQR)	1.75 (0.00–4.09)	0.00 (0.00–2.16)	1.79 (0.00–4.10)	0.229
HCV coinfection, n (%)	131 (31.2)	6 (75.5)	107 (30.2)	*0.013*
HBV coinfection, n (%)	21 (5.8)	1 (12.5)	20 (5.65)	0.383
AST U/L, median (IQR)	26 (20–38)	58 (29–92)	26 (20–37)	*0.041*
ALT U/L, median (IQR)	28 (18–50)	82 (37–122)	27 (18–49)	*0.047*
ARV backbone, n (%)				
ABC	32 (8.8)	1 (12.5)	31 (8.8)	0.311
AZT/DDI/D4T	223 (61.6)	7 (87.5)	216 (61.0)	
TDF	86 (23.7)	0 (0.0)	86 (24.3)	
Others	21 (5.8)	0 (0.0)	21 (5.9)	

Abbreviations: *n* number, *yrs*. years, *IQR* Inter Quartile Range, *MSM* Man how have sex with man, *IVDUs* Intra venous drug users, *BMI* Body Mass Index, *cps* copies, *ABC* abacavir, *TDF* tenofovir diproxil fumarate. *p-values are for χ2 or Fisher’s exact test and Mann-Whitney test

According to RUCAM, the 8 cases of NVP-induced liver toxicity were classified as hepatocellular injury and the likelihood of NVP-induced liver toxicity resulted possible for 3 patients and probable for 5 patients as shown in Table 2. A brief narration for each of the 8 cases is reported in Table 3.

**Table 2:** Updated RUCAM for the nevirapine-induced hepatocellular injury with the total scores for each patient

RUCAM items	Pt 1	Pt 2	Pt 3	Pt 4	Pt 5	Pt 6	Pt 7	Pt 8
1. Time to onset from the beginning of the drug • 5–90 days (rechallenge: 1–15 days) (+ 2) • < 5 or > 90 days (rechallenge: > 15 days) (+ 1) Alternative: Time to onset from cessation of the drug • ≤15 days (except for slowly metabolized chemicals: > 15 days) (+ 1)	+ 2	+ 2	+ 2	+ 2	+ 2	+ 2	+ 2	+ 2
2. Course of ALT after cessation of the drug • Percentage difference between ALT peak and N • Decrease ≥50% within 8 days (+ 3) • Decrease ≥50% within 30 days (+ 2) • No information or continued drug use (0) • Decrease ≥50% after the 30th day (0) • Decrease < 50% after the 30th day or recurrent increase (− 2)	+ 2	+ 2	+ 2	+ 2	+ 2	+ 3	+ 2	0
3. Risk factors • Alcohol use (current drinks/d: > 2 for women, > 3 for men) (+ 1) • Alcohol use (current drinks/d: ≤2 for women, ≤3 for men) (0) • Age ≥ 55 years (+ 1) • Age < 55 years (0)	0	0	+ 1	0	+ 1	+ 0	0	0
4. Concomitant drug(s) • None or no information (0) • Concomitant drug/herb with incompatible time to onset (0) • Concomitant drug/herb with compatible or suggestive time to onset (1) • Concomitant drug/herb known as hepatotoxin and with compatible or suggestive time to onset delete marking right side above (− 2) • Concomitant drug/herb with evidence for its role in this case (positive rechallenge or validated test) (− 3)	0	0	− 2	0	0	0	0	0
5. Search for alternative causes Tick if negative Tick if not done Group I (7 causes) • HAV: Anti-HAV-IgM • Hepatobiliary sonography / colour Doppler • HCV: Anti-HCV, HCV-RNA • HEV: Anti-HEV-IgM, anti-HEV-IgG, HEV-RNA • Hepatobiliary sonography/colour Doppler sonography of liver vessels/endosonography/CT/MRC • Alcoholism (AST/ALT ≥2) • Acute recent hypotension history (particularly if underlying heart disease) Group II (5 causes) • Complications of underlying disease(s) such as sepsis, metastatic malignancy, autoimmune hepatitis, chronic hepatitis B or C, primary biliary cholangitis or sclerosing cholangitis, genetic liverdiseases • Infection suggested by PCR and titer change for - CMV (anti-CMV-IgM, anti-CMV-IgG) - EBV (anti-EBV-IgM, anti-EBV-IgG) - HSV (anti-HSV-IgM, anti-HSV-IgG) - VZV (anti-VZV-IgM, anti-VZV-IgG) Evaluation of groups I and II • All causes-groups I and II—reasonably ruled out (+ 2) • The 7 causes of group I ruled out (+ 1) • 6 or 5 causes of group I ruled out (0) • Less than 5 causes of group I ruled out (− 2) • Alternative cause highly probable (− 3)	0	0	0	0	−2	+ 1	0	0
6. Previous hepatotoxicity of the drug • Reaction labelled in the product characteristics (+ 2) • Reaction published but unlabelled (+ 1) • Reaction unknown (0)	+ 2	+ 2	+ 2	+ 2	+ 2	+ 2	+ 2	+ 2
7. Response to unintentional reexposure • Doubling of ALT with the drug/herb alone, provided ALT below 5 N before reexposure (+3) • Doubling of ALT with the drug(s)/herb(s) already given at the time of first reaction (+ 1) • Increase of ALT but less than N in the same conditions as for the first administration (−2) • Other situations (0)	0	0	0	0	0	0	0	0
Total	+ 6	+ 6	+ 5	+ 6	+ 5	+ 8	+ 6	+ 4

Abbreviations: *pt*. Patient, *ALT* Alanine aminotransferase, *AST* Aspartate aminotransferase, *CMV* Cytomegalovirus, *CT* Computer tomography, *EBV* Epstein Barr virus, *HAV* Hepatitis A virus, *HBc* Hepatitis B core, *HBsAg* Hepatitis B antigen, *HBV* Hepatitis B virus, *HCV* Hepatitis C virus, *HEV* Hepatitis E virus, *HSV* Herpes simplex virus, *MRC* Magnetic resonance cholangiography, *N* upper limit of the normal range, *RUCAM* Roussel Uclaf Causality Assessment Method, *VZV* Varicella zoster virus

Total score and resulting causality grading: ≤0, excluded; 1–2, unlikely; 3–5, possible; 6–8, probable; and ≥ 9, highly probable

**Table 3:** Clinical characteristics of the 8 cases of NVP-induced liver injury. ^*^HLAB5701 tested absent

	Pt 1	Pt 2	Pt 3	Pt 4	Pt 5	Pt 6	Pt 7	Pt 8
HCV coinfection	no	yes	yes	yes	yes	no	yes	yes
ARV status	Experienced	Experienced	Experienced	Experienced	Naive	Experienced	Experienced	Experienced
Concomitant ARV	d4T + 3TC	d4T + 3TC	ABC^*^ + 3TC	AZT + 3TC	AZT + 3TC	d4T + ddi	AZT + 3TC	d4T + ddi
NVP exposure before treatment interruption (days)	28	61	29	28	58	28	50	38
Concomitant medication	none	none	Vitamin D and folinic acid	none	Phenobarbital and alprazolam	none	None	Folinic acid
Symptoms	Nausea and severe weakness	none	Nausea	none	Weakness	none	Nausea	None
Required hospitalization	yes	no	yes	no	no	no	no	no
Outcome	Recovered without sequelae	Recovered without sequelae	Recovered without sequelae	Recovered without sequelae	Recovered without sequelae	Recovered without sequelae	Recovered without sequelae	Recovered without sequelae
RUCAM	Probable	Probable	Possible	Probable	Possible	Probable	Probable	Possible

Abbreviations: *Pt* Patient, *HCV* Hepatitis C virus, *ARV* Antiretroviral, *d4T* Stavudine, *3TC* Lamivudine, *ABC* Abacavir, *AZT* Zidovudine, *ddi* Didanosine, *RUCAM* Roussel Uclaf Causality Assessment Method

Distribution of different genotypes of ABCB1 rs1045642, CYP2B6 rs3745274 and CYP3A4/A5 combined are shown in Table 4. A statistical significant difference was observed in patient with and without NVP-induced hepatotoxicity according to ABCB1 rs1045642 genotypes (*p = 0.019*).

**Table 4:** Disposition of polymorphisms involved in nevirapine metabolism

	Total	Hepatotoxicity	No hepatotoxicity	p*
	*n* = 362	*n* = 8	*n* = 354	
ABCB1 c.3435/rs1045642, n (%)				*0.019*
CC	86 (23.8)	5 (5.8)	81 (94.2)	
CT	178 (49.2)	1 (0.6)	177 (99.4)	
TT	98 (27.0)	2 (2.0)	96 (98.0)	
CYP2B6 c.516/rs3745274, n (%)				0.706
GG	196 (54.1)	6 (3.1)	190 (96.9)	
GT	141 (39.0)	2 (1.4)	139 (98.6)	
TT	25 (6.9)	0 (0.0)	25 (100.0)	
CYP3A4/A5 **, n (%)				0.602
*Extensive*	58 (16.1)	0 (0.0)	58 (100.0)	
*Intermediate*	270 (74.5)	8 (3.0)	262 (97.0)	
*Poor*	25 (6.9)	0 (0.0)	25 (100.0)	
nd	9 (2.5)	0 (0.0)	9 (100.0)	

Abbreviations: *n* number, *nd* not determined, *ABCB* ATP Binding Cassette Subfamily B, *CYP* Cytochrome P450 enzyme

*χ2 test ** CYP3A4*22/rs35599367 and CYP3A5*3/rs776746 combined genotypes for comprehensive functional evaluation [33, 34]

In univariate analysis (Table 5), male gender (OR = 0.19 95%CI 0.04–0.94; *p = 0.042*), HCV coinfection (OR = 6.93 95%CI 1.38–34.87; *p = 0.019*), AST (OR = 1.02, 95%CI 1.01–1.03; *p = 0.008*) and ALT (OR = 1.01, 95%CI 1.00–1.02; *p = 0.015*) median level at the beginning of NVP have been associated with an increased risk of severe NVP-induced liver toxicity. On the other hand, genotypes CT/TT of ABCB1 rs1045642 (OR = 0.18, 95%CI 0.04–0.76; *p = 0.020*) and greater value of body mass index (OR = 0.7 95%CI 0.51–0.94, p = 0.02) showed a protective effect.

**Table 5:** Backward logistic regression of factors involved in nevirapine induced liver toxicity

	OR (95%CI)	*p*	aOR (95%CI)	*p*
Male vs Female	0.19 (0.04–0.94)	*0.042*	0.27 (0.06–1.30)	0.102
Age (× 1 year more)	0.96 (0.88–1.04)	0.293	–	
MSM vs HE	0.24 (0.01–4.51)	0.338	–	
IVDUs vs HE	2.20 (0.58–8.41)	0.247	–	
Other vs HE	1.36 (0.06–29.16)	0.844	–	
Caucasian vs Non-Caucasian	0.67 (0.08–5.64)	0.714	–	
BMI (× 1 more)	0.70 (0.51–0.94)	*0.020*	0.72 (0.52–1.00)	0.050
Previous AIDS	0.67 (0.08–5.56)	0.713	–	
Previous therapy duration (× 1 year more)	1.05 (0.90–1.23)	0.518	–	
CD4 200–500 cell/μL vs < 200 cell/μL	1.17 (0.05–25.7)	0.920	1.51 (0.07–32.23)	0.790
CD4 > 500 cell/μL vs < 200 cell/μL	3.97 (0.21–74.51)	0.357	8.12 (0.42–156.90)	0.166
HIV-RNA (× 1 log10 more)	0.80 (0.54–1.18)	0.260	–	
AST (× 1 more)	1.02 (1.01–1.03)	*0.008*	1.01 (0.99–1.03)	0.144
ALT (× 1 more)	1.01 (1.00–1.02)	*0.015*	–	
HCV coinfection	6.93 (1.38–34.87)	*0.019*	8.00 (1.27–50.29)	*0.027*
HBV coinfection	2.39 (0.28–20.36)	0.427	–	
ARV Backbone: AZT/DDI/D4T vs ABC	0.73 (0.12–4.47)	0.731	–	
ARV Backbone: TDF vs ABC	0.12 (0.01–3.141)	0.204	–	
ARV Backbone: Other vs ABC	0.49 (0.02–13.47)	0.672	–	
ABCB1 rs1045642 CT/TT vs CC	0.18 (0.04–0.76)	*0.020*	0.10 (0.02–0.47)	*0.004*

Abbreviations: *OR* Odds Ratio, *aOR* adjusted Odds Ratio, *CI* confidence interval, *HE* Heterosexual, *MSM* Man how have sex with man, *IVDUs* Intra venous drug users, *BMI* Body Mass Index, *cps* copies, *ABC* abacavir, *TDF* tenofovir diproxil fumarate, *BMI* Body Mass Index, *ABCB* ATP Binding Cassette Subfamily B

In the multivariate logistic regression model (Table 5), HCV coinfection was confirmed to be independently associated with a higher risk of developing liver toxicity (aOR = 8.00, 95%CI 1.27–50.29; *p = 0.027*), whereas ABCB1 CT/TT genotypes (aOR = 0.10, 95%CI 0.02–0.47; *p = 0.004*) has been associated with a lower risk; a higher body mass index (aOR = 0.72, 95%CI 0.519–1.000; *p* = 0.050) has been barely related to a lower risk (Table 5). On the contrary, the association between gender, baseline AST levels and NVP-induced liver toxicity wasn’t confirmed in the final multivariate model.

## Discussion

In this study conducted in a monocentric cohort of HIV-1 positive patients exposed to NVP we observed, during the first three months of treatment, an incidence of severe liver toxicity of 2.2%. This finding is similar to that reported by other cohorts when NVP was used in *experienced* patients [10, 22]. We did not observe a significant association between the development of hepatotoxicity and high CD4 cells count at NVP start, supporting the observation of low frequency of NVP induced liver toxicity in *experienced* patients [10]. The mechanisms involved in the development of severe hepatotoxicity are not well explained and it could be that in patients never exposed to antiretroviral therapy immune-mediated process leading to immune-reconstitution could elicit the development of liver toxicity [23]. On the contrary, in experienced patients with a stable immune-virological situation, hepatotoxicity could be driven by a direct effect of the drug in susceptible patients [24]. We confirm previous findings supporting the importance of HCV coinfection as independent factor associated to the development of NVP-related liver toxicity; HCV infection could play a direct role causing liver injury and also could interfere with the metabolism of the drug [8, 11, 25]. The enhanced risk of development hepatotoxicity in HCV coinfected patients treated with NVP seems to be independent from NVP plasmatic concentrations, since comparable NVP concentration are observed in patients with and without HCV coinfection [26].

A significant correlation between low value of body mass index (< 18.5) such as for increased NVP plasma concentration and increased risk for hepatotoxicity has been previously reported [12, 18], in our study we observed a trend of body weight in predisposing to NVP-hepatotoxicity albeit not confirmed in the multivariate model.

In accordance with previous studies, we did not found a statistically significant association between polymorphisms in CYPs genes (CYP2B6, CYP3A4, CYP3A5) and the development of severe hepatotoxicity. Although these genes are involved in NVP metabolism [27] and their functional variants may significantly affect NVP plasma concentrations [28–30], their role in predisposing NVP induced hepatotoxicity remains unclear [19, 31].

The ABCB1 gene encodes for P-glycoprotein, one of the most important efflux pomp involved in the transport of both NVP and efavirenz and a modification of this protein could determine an alteration in the intracellular concentration of these drugs [32].

Interestingly, the functional variant c.3435 C > T of ABCB1 gene was associated with an increases risk for severe liver toxicity in a previous study conducted by Hass et al. in South-Africa [19]. Moreover, the variant ABCB1 c.3435 C > T resulted protective for NVP-associated hepatic adverse events in another study conducted in Mozambique [20]. No statistically significant association between ABCB1 c.3435 C > T and NVP adverse events was found in another study conducted by Yuan J et al. in an ethnic mixed population. However, these authors found a significant association between ABCB1 c.3435 C > T variant and hepatic adverse events among Africans but not Asians or Caucasians, despite these latter groups showed increased T-allele frequencies [31].

Overall our study, which was conducted on a prevalently European Caucasian population, disagrees with the study by Yan et al. On the contrary it supports the protective role of T allele of ABCB1 rs1045642 for NVP-hepatotoxicity evidenced by Hass et al. and Ciccacci et al.

The present study has some limitations. In particular, due to the retrospective design we cannot exclude the presence of possible bias related to loss of data. A sub-optimal performance of RUCAM, which is more fitted for a prospective patient evaluation, could be hypothesized because of the retrospective nature of the study. Moreover, if on one hand the limited number of NVP induced hepatic adverse events supports the good safety profile of this drug in ART-experienced patients, on the other hand our study could not exclude that the prevalence of the investigated polymorphisms could be driven by chance. Moreover, the prevalence of Caucasian ethnicity limited the comparison of our findings between different ethnic groups.

## Conclusion

Beyond to clinical conditions well known to drive the development of hepatotoxicity during NVP treatment, i.e. HCV coinfection and body mass index, pharmacogenomic profiles could also play a role in this phenomenon. Our results suggest the independent role of ABCB1 rs1045642 as a predictive marker of severe liver toxicity related to NVP. Further validation studies, to assess possible clinical application of this marker in countries in which NVP is still widely used and/or in patients with other risk factors for nevirapine related toxicity, are warranted.

## Abbreviations

ABCB: ATP Binding Cassette Subfamily B
aOR: Adjusted Odds Ratio
ART: Antiretroviral treatment
CI: Confidence interval
CYP: Cytochrome P450 enzyme
HIV-1: Human immunodeficiency virus type 1
IQR: Inter quartile range
NVP: Nevirapine
OR: Odds Ratio
RUCAM: Roussel Uclaf Causality Assessment Methods
SNPs: Single nucleotide polymorphisms
